# 10 tips for a transition clinic from paediatric to adult nephrology

**DOI:** 10.1093/ckj/sfaf347

**Published:** 2025-11-14

**Authors:** Constantina Chrysochou, Alexander Hamilton, Klebert Tembe, Lars Pape, Nele Kanzelmeyer, Alexander Woywodt

**Affiliations:** Donal O’Donoghue Renal Research Centre, Salford Royal Hospital, Northern Care Alliance NHS Foundation Trust, Salford, UK; Division of Cardiovascular Sciences, Faculty of Biology, Medicine & Health, University of Manchester, Manchester, UK; Royal Devon University Healthcare NHS Foundation Trust, Exeter, UK; Salford Royal Hospital, Northern Care Alliance NHS Foundation Trust, Oldham, UK; Department of Pediatrics II, Essen University Hospital, University of Duisburg-Essen, Essen, Germany; Department of Paediatric Kidney, Liver, Metabolic and Neurological Diseases, Hannover Medical School, Hannover, Germany; Lancashire Teaching Hospitals NHS Foundation Trust, Preston, UK

**Keywords:** children and young people, multidisciplinary, neurodevelopment, social prescribing, transition

## Abstract

The transition from paediatric to adult renal care is a challenging time for children and young people (CYP). For them, this is a period of seismic change overall and a key step during their journey from parental support to independence. Improving outcomes in paediatric care is resulting in a growing population of CYP who require transition and individualised care planning. The point of transfer to adult services is also a risk escalator to long-term health and it is important to avoid disengagement. Awareness of neurodevelopmental biology and its effects on decision-making is a cornerstone to understanding the behaviour of CYP during this time. There is good evidence to suggest that a holistic approach to transition, rather than simply a formal transfer of care, improves patients’ understanding and ability to self-care, and in so doing reduces morbidity and mortality. A coordinated process of transition involves empowerment and shared decision-making and starts in paediatric care aided by transition toolkits. In the absence of a structured transition, CYP often present to adult services unplanned and in a crisis situation. CYP presenting directly to adult services are a particularly vulnerable group with unmet needs due to missing out on a formal transition process. Local innovation, regional initiatives and national policy can help reduce variation and ensure access to good-quality transition care and clinics. Setting up such transition clinics requires careful planning, a multidisciplinary approach and adequate long-term funding. We provide a brief toolkit to set up, run and develop a structured transition service.

## INTRODUCTION

Chronic kidney disease (CKD) in children shares mechanisms and pathophysiology with kidney disease in adults but there are significant differences and some have considered it a stand-alone disease entity [1]. Children and young people (CYP) with CKD requiring dialysis or transplantation face numerous challenges that differ from those faced by their adult counterparts, namely negotiating puberty, with its biological, psychological and developmental changes. Peer pressure, risk-taking behaviour and social media can influence trajectories significantly. Symptoms (such as polyuria, fatigue, itching and nausea to name a few), a high pill burden and invasive procedures can compound anxiety. CYP have a higher morbidity and mortality rate than their healthy peers [1]. CYP are also disadvantaged by an increased risk of cognitive impairment that impacts academic attainment [2].

Transition is defined as a ‘purposeful, planned process that addresses the medical, psychosocial and educational needs’ of CYP as they advance from a paediatric and family-centred to an adult, individual-focused model of care. There is now ample evidence for a structured approach to transition and for dedicated transition clinics, not just in nephrology [3], but in many fields such as genetics [4], rheumatology [5] and respiratory medicine [6, 7]. However, this evidence is yet to translate into clinical practice and the uptake and quality of dedicated transition clinics has remained variable [8].

Transition often represents a challenging time for CYP and their caregivers alike [2] due to a perceived loss of an established relationship with the paediatric team. This can be further compounded by a lack of coordination, resources and support in either or both paediatric and adult services [9]. As a result, many young people are disengaged or lost to follow-up when transferred to an adult system. In one study of young transplant patients, as many as 8 of 20 patients lost their graft within 36 months of transfer to adult nephrology [10]. Lack of structured transition thereby leads to resource impacts and cost on health services through avoidable complications. In addition, transitioning through services often occurs in pivotal periods of education, e.g. examinations and moving to college or university. Along with several factors, employability can be impacted, with 15 times greater odds of being unable to work due to health in CYP with kidney failure [11]. These long-term consequences and enduring societal costs are rarely considered.

Challenges also exist for nephrologists, and setting up a dedicated transition clinic is not trivial. First, there is a substantial multidisciplinary team to set up, fund and coordinate. Second, young adults have distinct developmental and separate biopsychosocial needs. Third, communication with CYP and their families is typically different in style from adult nephrology. However, plentiful opportunities also exist: innovative ‘co-created’ clinic approaches and novel educational packages can be created. CYP are often very information technology literate, which can help with virtual care and telemedicine.

**Figure 1:**
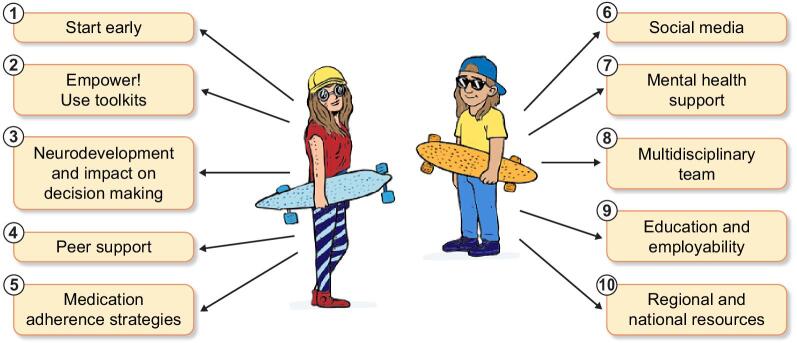
Ten tips for a transition clinic from paediatric to adult nephrology.

As a group of adult and paediatric nephrologists and supported by one of our former transition clinic patients, we pooled collective experience and best practice to provide 10 practical tips for colleagues who wish to set up a dedicated transition service (Fig. 1). For the purposes of this article, we refer to CYP as encompassing ages 12–24 years. This honours legal and safeguarding definitions of a child being a person <18 years of age and ‘young people’ to cover ages 16–24 years.

## TIP 1: START THE TRANSITION PROCESS EARLY AND AIM FOR CONTINUITY OF CARE

An early start to the transition process is crucial to ensure time and ‘headspace’ to assimilate information, continuity of care and favourable long-term outcomes. Stabile *et al.* [12] reported abrupt transfers and inadequate cross-team handovers as reasons for becoming ‘lost to follow-up’. Conversely, addressing CYP individually and in an adult manner with written communication of the transition process inspires confidence in the process for both healthcare givers and recipients. Both European and US transition programs advocate discussions regarding the concept and timing of transition with patients and their families starting at the age of 12–14 years [13]. This allows sufficient time to establish an individual transition plan, educate the patient and his/her family, gain confidence in self-management, address anxieties and repeatedly review their knowledge and skills. For patients struggling with adherence, alternative medications can be arranged in advance [14]. Continuity of care by a named consultant or familiarity with the adult team members while in paediatric care, along with clear messages [10], help avoid miscommunication and medical complications [15].

## TIP 2: TRANSITION IS ABOUT EMPOWERMENT—UTILISE TOOLKITS!

Every patient journey is unique. Toolkits provide a useful framework to guide transition and monitor progress in an objective manner (Table 1). They open conversations on challenging topics, e.g. risk-taking and sexual health. They benefit patients by exploring health, emotional, social, mental, education and vocational needs, which may need support during and following transition to adult services. International initiatives [7, 16] are often generic and not limited to one specialty. Good examples include the nurse-led ‘Movin’ on Up’ for adolescents in the USA [17] and the ‘Ready Steady Go Hello’ program in the UK [18]. The latter has a follow-on toolkit for use in adult medicine and can be utilised for young adults presenting directly to adult services. Similar programs exist in Canada [19], Australia and continental Europe [20].

**Table 1:** Examples of transition toolkits used in CKD offering free online resources.

Transition Toolkits that offer a structured approach to Transition
ACP Pediatric to Adult Care Transitions Toolkit [58]
Adolescents and young adult transition model in Africa [59]
Developmentally Appropriate Healthcare Toolkit [60]
Got Transition’s Six Core Elements of Health Care Transition™ 3.0^a^ [52]
Ready Steady Go Hello [18]^b^
Ten Steps to Transition [61]
Together for Short Lives [62]
Toolkit on the Transition of Unaccompanied Migrant Children to Adulthood [63]

^a^Available in Spanish.

^b^Available in several languages.

Toolkits prompt patient-centred interventions and can be adapted to the individual accordingly:

- Asking CYP to complete a toolkit questionnaire ahead of an appointment to ensure issues important to them aren’t missed or felt less important [21].
- Using ‘shared decision-making’ style questions, e.g. ‘What are your options?’, ‘What are the benefits and harms of each choice?’, ‘How do you get support to make a decision that is right for you?’, ‘What worries you most about your health or treatment?’, ‘How does your health affect your day-to-day life?’
- Writing letters directly to patients in a language they can understand [22].
- Providing CYP and parents/caregivers with contact details of the team.
- Providing written or video tours of the adult service environment [23].
- Supporting parents or caregivers during the transition process, whose role and fears about transition can be underappreciated [20]. They may worry about their child’s preparedness for greater independence and fear changes in healthcare providers and unfamiliar systems leading to potential gaps in the quality of care and negative health outcomes. Overcoming these fears requires proactive planning, hence starting transition early and ensuring open communication between paediatric and adult providers. Graduating through stages of a transition program to gradually shift responsibility to the young adult, such as encouraging more responsibility, self-advocacy and solo visits, helps provide reassurance and confidence to patients and their parents or caregivers alike.

Policies and toolkits can support healthcare professionals by allowing identification of best practices and benchmarking, by highlighting variation and by providing much-needed evidence for service development and funding. Toolkits are of particular value when considering core outcomes for quality improvement or research. The Standardised Outcomes in Nephrology initiative has developed outcome sets based on the shared priorities of patients, caregivers, clinicians and other relevant stakeholders, including one for CYP [24].

## TIP 3: LEARN ABOUT NEURODEVELOPMENT AND ITS INFLUENCE ON DECISION-MAKING AND RISK-TAKING

During the transition, CYP are expected to assume increasing responsibility for managing their own healthcare while the roles of parents and caregivers are slowly decreasing. However, this expectation arises at a time when brain development remains incomplete: the brain matures along a back-to-front trajectory [25] and subcortical regions like the limbic system, which are involved in emotion and reward processing, mature earlier. In contrast, the prefrontal cortex—responsible for planning, impulse control and decision-making—continues developing well into the mid-20s [26]. The imbalance between emotion/reward systems and cognitive control networks helps explain why adolescents tend to prefer immediate rewards over delayed gratification [27], take more risks [28] and show inconsistent adherence to health recommendations. For example, a teenager may intellectually understand the risks of not taking medication but still prioritize short-term immediate pleasure over long-term health outcomes. Executive functions, including planning and working memory, are often underdeveloped, and heightened emotional reactivity can interfere with rational decision-making. Additionally, while adolescents may seek greater autonomy, they may lack self-regulation. A sense of perceived invulnerability can further drive risk-taking behaviours that lead to non-adherence or non-attendance [29].

All healthcare providers may need to consider their learning needs in terms of knowledge and skills around these important developmental processes [30]. Healthcare providers are also advised to use developmentally appropriate communication strategies, to consciously make efforts to understand what matters most to patients, to support incremental autonomy by gradually increasing self-management responsibilities and to provide structured reminders with motivational interviewing. It is essential to recognize that cognitive maturity—such as understanding one’s illness—does not necessarily equate to behavioural maturity.

Current transition models often rely primarily on chronological age rather than neurocognitive readiness, which can result in mismatched expectations. These challenges are even more pronounced for adolescents with neurodevelopmental disorders. There is a pressing need for neurodevelopmentally informed transition frameworks that align support and responsibilities with an individual’s cognitive and emotional maturity. At the same time, it should be acknowledged that continued brain maturation into the mid-20s is normal. Society commonly considers 18-year-olds capable of voting, driving, attending university or entering the workforce—so why then should self-care and adherence be the exceptions?

## TIP 4: SIGNPOST PEER SUPPORT STRUCTURES AND FOSTER A SENSE OF COMMUNITY

Difficulties in social inclusion, loneliness and an absence of normalcy are key stressors for social functioning in CYP with chronic conditions [31]. Peer support offers a unique way for individuals to navigate the challenges of chronic health conditions with others who have similar experiences. Members can share coping strategies and discuss sensitive issues in a confidential environment free of medical judgement and stigma [32]. CYP undergoing transition often face additional stigma through being excluded from normative activities during school and frequent medication regimens can lead to further stigma [33]. Furthermore, CYP with kidney disease may be vulnerable to bullying stemming from changes in physical appearance, such as reduced growth as well as weight gain and a cushingoid appearance from steroid exposure [34]. These challenges are rarely communicated to healthcare professionals. Peer support should also be provided through virtual interventions in order to maximise access and facilitate attendance [35]. In the UK, the Young Adult Kidney Group, supported by Kidney Care UK, was established in 2016 and has expanded to multiple social media platforms [36]. While only offering limited spaces, the group hosts an annual residential ‘summer camp’–style weekend. The camp features icebreaker sessions, cooking classes and outdoor activities such as rock climbing, bushcraft and archery.

## TIP 5: PROVIDE SUPPORT FOR MEDICATION ADHERENCE CHALLENGES

Adherence, be it regarding medications, regular blood and urine tests and investigation or outpatient appointments, is a key issue for patients transitioning from paediatric to adult nephrology. Studies have estimated 44% of all graft losses and 23% of late acute rejection episodes may be due to non-adherence [37]. It is also important to acknowledge more subtle forms of non-adherence may not prompt severe acute rejection but contribute to a loss of function long term. Across all age groups, multiple factors can lead to non-adherence [38]. Common causes include psychological factors and mental health issues, social and behavioural factors and those relating to medical factors, e.g. difficulties with communication due to visual impairment. Practical tips to improve adherence are discussed in detail elsewhere [38]. Examples include simpler dosing regimens, setting reminders or timers on phones, a pharmacist to support drug education, peer support, discrete dosette boxes, point-of-care testing and investigations conducted closer to home. It is important to avoid stigma or reflex labelling of patients as non-adherent. However, issues around adherence should be clearly communicated during the handover of care. An open-minded and holistic approach that focuses on providing support should be used and provider continuity is also important.

## TIP 6: UTILISE SOCIAL MEDIA FOR EDUCATION

The daily lives of young people and their caregivers are interwoven with digital technology and their online identity is very important to many of them. The internet and social media have been rapidly adopted by patients and caregivers as means not just to source information but also to share lived experiences in the forms of content creation and to seek emotional support from peers. The integration of social media in chronic care management [39, 40] is an important tool to disseminate health information and support decision-making. This comes with the caveat that content should be considered a reliable source and updated over time. Sourced information should be discussed with doctors to ensure relevance for the individual and that misinformation is tackled. Popular resources include Rare Diseases Europe (EURORDIS) [41], a non-governmental patient-driven alliance of >1000 rare disease patient organisations from 74 countries. Targeted information is easily navigated and divided by disease type [41]. The British Association for Paediatric Nephrology collaboration with Kidney Care UK launched InfoKid [42], an evidence-based platform to promote awareness around CKD. The platform has produced educational materials about the root causes of disease, signs and symptoms and disease treatment in the form of e-leaflets and videos through YouTube. Similar resources are available elsewhere.

## TIP 7: FOCUS ON MENTAL HEALTH AND IDENTIFY RESOURCES TO PROVIDE SUPPORT

The 2018 Surveying Patient Experiencing Young Adult Kidney Failure (SPEAK) study [43] recruited 976 young adults, comprising both those with transplants and those receiving dialysis treatment. Compared with the general population, young adults on renal replacement therapy were less likely to be in a relationship or have children. They were also more likely to live in the family home, receive no income and be unable to work due to health. They had poorer quality of life, worse well-being and twice the likelihood of a psychological disturbance [odds ratio 2.7 (95% confidence interval 2.0–3.7), *P* < .001]. Mental health problems were negatively associated with well-being and medication adherence [45] and were possibly underrecognised. The study used the 12-item General Health Questionnaire to assess psychological problems, which predominantly focuses on depression and anxiety. In a 5-year follow-up study of the original cohort [11], the nature of the psychological issues was clarified using the nine-item Patient Health Questionnaire and the seven-item Generalised Anxiety Disorder scale. Overall, the prevalence of anxiety and depression were found to be 40% and 35%, respectively. This study took place during the coronavirus disease 2019 pandemic, which may have impacted the findings. The SPEAK study findings suggest that routine screening of CYP for common mental health difficulties is warranted. Potential outcomes include active monitoring within the transition clinic, preventative measures and referral to counselling services or to specialised renal psychology services. Variation in psychosocial support for people with kidney disease [44] is one of the main barriers towards this goal, with a quarter of kidney units in the UK reporting no access to renal psychology in a 2017 study [45].

## TIP 8: DEVELOP A MULTIDISCIPLINARY TEAM FOR A HOLISTIC APPROACH

**Figure 2:**
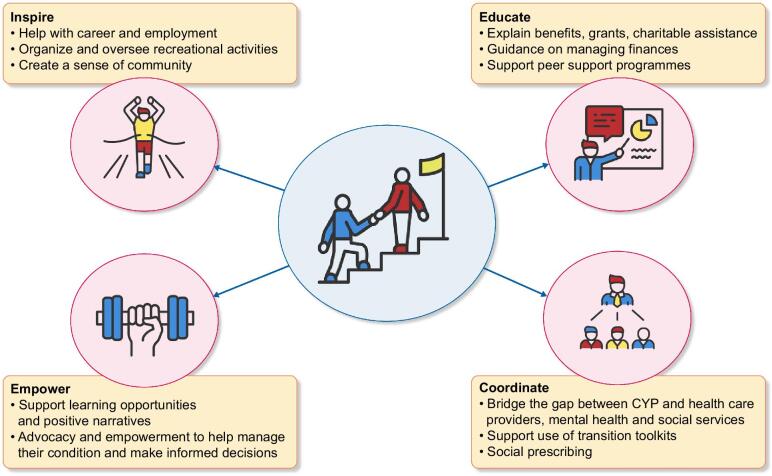
Benefits of a youth worker.

To ensure a successful transition, a multidisciplinary team should be set up to include ideally three separate disciplines [46]. This diversity in skills enables comprehensive, patient-centred care [15]. An effective multidisciplinary team usually includes paediatricians and adult physicians, paediatric and adult nurses, allied health professionals such as psychologists or social workers, youth workers, pharmacists and educators. Youth workers in particular provide a vital connection between CYP and healthcare professionals, foster engagement, social prescribing [47] and networking and employability, among others (Fig. 2). Multidisciplinary transition programs, which are co-created with patients, improve patient satisfaction and adherence [22, 48].

## TIP 9: THINK OF EDUCATION AND EMPLOYABILITY

Education and employment are not merely background factors, they actively shape transition readiness, autonomy and access to healthcare. CYP with chronic health conditions experience executive function deficits, school absenteeism and academic delays. Despite high rates of secondary school graduation (≈97%), ≈20% of young adults with CKD are unemployed or reliant on disability benefits by age 22 [33].

Education and employment also serve as scaffolding for the development of cognitive and behavioural skills and determine access to adult healthcare systems through financial stability, insurance coverage and workplace benefits. A qualitative study of young adults on renal replacement therapy demonstrated that social support within schools and workplaces was essential to maintaining engagement and independence [49]. A lack of support in education or work can therefore undermine transition success. However, CYP may also fear stigma when disclosing a medical condition in order to access additional support. Most transition programs remain predominantly health-focused, often neglecting the educational and vocational dimensions that are integral to long-term well-being. Effective transition planning should therefore integrate educational and vocational services [50].

Programs such as the Educational Video Series developed by the North West Kidney Network [51] were designed specifically for teachers, teaching assistants, school nurses and other education staff. These videos help build understanding and competence in supporting CYP with kidney disease in school settings and could serve as a model for other regions. The development of transition programs should prioritize participatory design involving adolescents themselves, to better integrate academic, vocational and psychosocial objectives alongside medical goals.

## TIP 10: IDENTIFY REGIONAL AND NATIONAL SOURCES OF SUPPORT AND FUNDING

Local initiatives for transition usually work better when embedded in a framework of national guidance and support. The ‘Got Transition’ approach in the USA [52] defines six core elements of transition and lists components of a structured transition process that is applicable regardless of disease or specialty. A similar framework of guidance exists in the UK [53] that defines high-quality transition care and also identifies priority areas for improvement. Three key principles within the Renal Services Transformation Programme transition element, consistent with the National Framework for Transition, were set out. These were ‘working together’, ‘providing the right care’ and ‘at the right time’. The actions outlined the importance of an integrated and cross-service provision approach with key metrics to monitor effectiveness and enable data collection.

In most other European countries, transition programs for renal patients are less well established, often due to lack of funding. Germany public healthcare, for example, only funds paediatric care until the 18th birthday [54] and specific transition services are generally not reimbursed. The Berlin Transition program is a notable exception, although a prospective randomized trial failed to show benefit [55]. Other programs such as ‘Between Youth Coaching’ [56] are available in Germany and a special transition workshop at a rehabilitation centre for children on dialysis or after transplantation is offered but financed privately [57].

To improve the situation, we would very much encourage international, collaborative initiatives with free resources allowing for validation and comparability of data. As an example, the UK ‘Ready Steady Go Hello’ transition program has been translated into several languages and can serve to kickstart new national transition [18] programs. Finally, it is also noteworthy that there are currently no international guidelines on this topic and the existence of such guidelines may help improve the situation further.

## CONCLUSION

Adolescents with CKD face unique and complex challenges in health, well-being, education and employment that are closely linked to cognitive, social and health-related factors. Adolescent brain development is marked by the early maturation of reward systems and the later maturation of cognitive control networks. This mismatch fosters heightened risk-taking and inconsistent decision-making. Transition programs in nephrology must expand beyond a narrow focus on managing clinical issues and ‘medical’ readiness. Instead, it is important to also consider individual biopsychosocial development, facilitate empowerment and think about educational support. Several transition toolkits exist to guide transition readiness and support a successful transition into adult care and life. Good-quality transition programs extend into early adulthood and ensure continuity along with a dedicated multidisciplinary team. National guidelines and workstreams can support the implementation of a high-quality transition program and reduce variation in care. We would like to encourage colleagues to share our experience, and we hope our 10 tips (Fig. 1) help in setting up, developing and growing transition services overall.
